# Zygomycosis in Two Hematologic Cases

**DOI:** 10.1155/2011/181782

**Published:** 2011-07-17

**Authors:** M. T. García-Romero, J. García-Méndez, R. Arenas, T. Ferrari-Carballo, J. Chanona-Vilchis, E. Cervera-Ceballos

**Affiliations:** ^1^Departments of Dermatology, Hospital General Dr. Manuel Gea González, 14080 Mexico City, Mexico; ^2^Departments of Infectious Diseases, Instituto Nacional de Cancerología, 14080 Mexico City, Mexico; ^3^Departments of Mycology, Hospital General Dr. Manuel Gea González, 14080 Mexico City, Mexico; ^4^Departments of Radiology, Instituto Nacional de Cancerología, 14080 Mexico City, Mexico; ^5^Departments of Pathology, Instituto Nacional de Cancerología, 14080 Mexico City, Mexico; ^6^Departments of Hematology, Instituto Nacional de Cancerología, 14080 Mexico City, Mexico

## Abstract

Zygomycosis are invasive mould infections, rarely diagnosed in hematologic patients. Most of the cases published are in patients with prolonged neutropenia, along with other risk factors such as the use of prior broad-spectrum antibiotics (including new antifungal agents, such as voriconazole), diabetes mellitus (with or without ketoacidosis), malnutrition, iron overload (with or without the use of deferoxamine). These infections have poor prognosis due to the involvement of vital anatomic structures and late diagnosis. Until recent years, the treatment was based on high doses of amphotericin B plus surgical debridement. Here we present two patients with hematologic diseases (one with leukemia, the second with aplastic anemia) with an impaired immune system and the diagnosis of zygomycosis. The survival of one of them was mainly due to early diagnosis and surgical debridement; unfortunately the second was misdiagnosed as an extensive ecchymosis due to thrombocytopenia and died with CNS involvement.

## 1. Introduction

Most invasive fungal infections occur in patients with hematologic malignancies, and the prevalence has increased steadily in recent years. This phenomenon is attributed to host defense impairment due to intensive cytotoxic chemotherapies, stem cell transplantation, myeloablative radiation therapy, and the use of corticosteroids, cyclosporine, or new immunosuppressive agents. Although *Candida* is the main fungal agent involved in invasive fungal infection (IFI), an increasing number of infections are caused by molds, mostly *Aspergillus spp*, but in the last 20 years other emerging fungal pathogens such as Zygomycetes have appeared with high mortality rates [1]. We present two similar cases with different evolutions.

## 2. Clinical Cases

**Figure 1:**
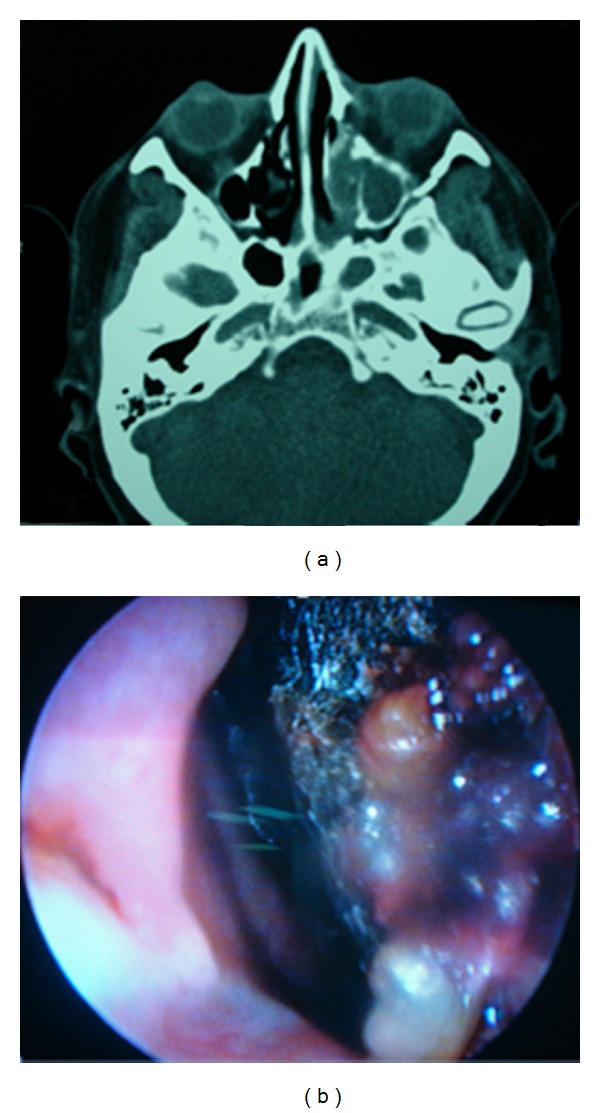
(a) CT scan of patient with invasion of left paranasal sinuses and orbit. (b) Necrotic sinus tissue found on endoscopy.

**Figure 2:**
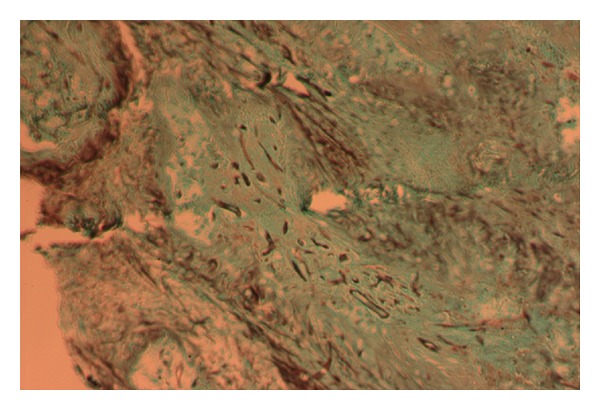
Biopsy with silver stain showing large pauciseptate hyphae invading tissue.

**Figure 3:**
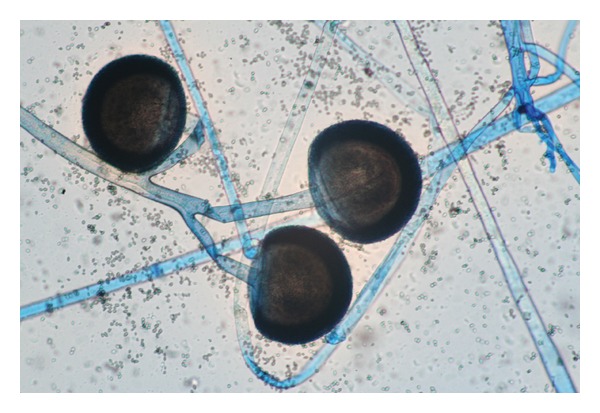
Sporangiophores with big sporangia, with no apophysis, rhizoids or stolones, compatible with *Mucor sp*.

Patient no. 1 was a 55-year-old male, referred to our hospital, with a 4-month history of fatigue, weight loss, anorexia, nocturnal diaphoresis, occasional vertigo, and recent several ecchymoses below both knees. On admission he was found pale, with multiple vesicles on lips and cheeks, and cervical lymphadenopathies (larger than 1 cm). The hemogram showed leukocytes 11.8/mm^3^, 10.6/mm^3^ lymphocytes (89%), 0.5/mm^3^ neutrophils (4%), hemoglobin level 10.6 g/dL, and 38/mm^3^ platelets. A blood smear was performed, describing blasts somewhat large with a varied morphology which was classified as Acute Lymphoblastic Leukemia (ALL) L2 according to French-American-British (FAB) classification. An additional diagnosis of herpes simplex virus infection was made. He was put on Cancer and Leukemia Group B (CALBG) scheme and intravenous acyclovir and hospitalized. Two weeks later he developed fever associated to profound neutropenia (100/mm^3^), and ceftazidime plus amikacin was added to the treatment according to Infectious Diseases Society of America (IDSA) guidelines. Four days later, due to persistent fever, vancomycin was added with incomplete defervescence. All cultures were negative, and no source of infection was identified. On day 14 of chemotherapy, he complained of periorbital pain at his left eye. He was found to have slight proptosis and conjunctival hemorrhage. Because of persistent fever, and the sinus maxillary clinical signs, a CT scan of the head was performed; it showed a solid image inside the left maxillary sinus, proptosis of the left eyeball, and destruction of the structures adjacent to the nose (Figure 1(a)). Following these findings, a nasal endoscopy was performed promptly, and it showed an extensive necrosis of the left medial turbinate (Figure 1(b)). An incisional biopsy report showed necrosis and hemorrhage with nonseptate hyphae 90° degree branched (Figure 2), and Mucor sp. was identified on culture (Figure 3) since the first procedure intranasal amphotericin was instilled. During a 4-week period, three nasal endoscopies were performed until necrotic tissue disappeared, along with antibacterial and antifungal therapies (amphotericin B 75 mg daily). Amphotericin B was discontinued after completing 1.0 gram, then switched to itraconazole. He was able to be continued on the chemotherapy protocol and ultimately recovered from neutropenia. He was kept on itraconazole during the following chemotherapy administrations, until he was declared free of disease. Until now, he is in remission of his ALL. 

**Figure 4:**
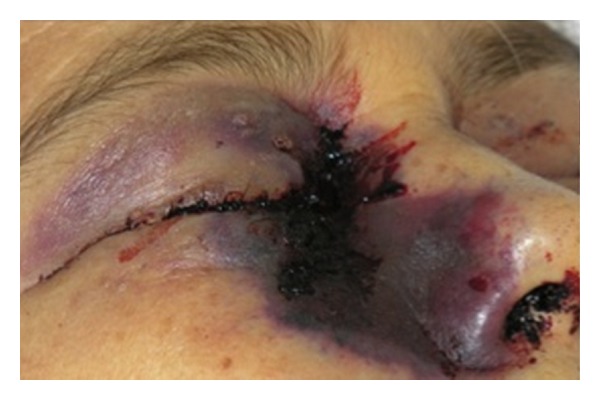
Aspect of patient with periorbital edema and ecchymosis of the surrounding skin.

**Figure 5:**
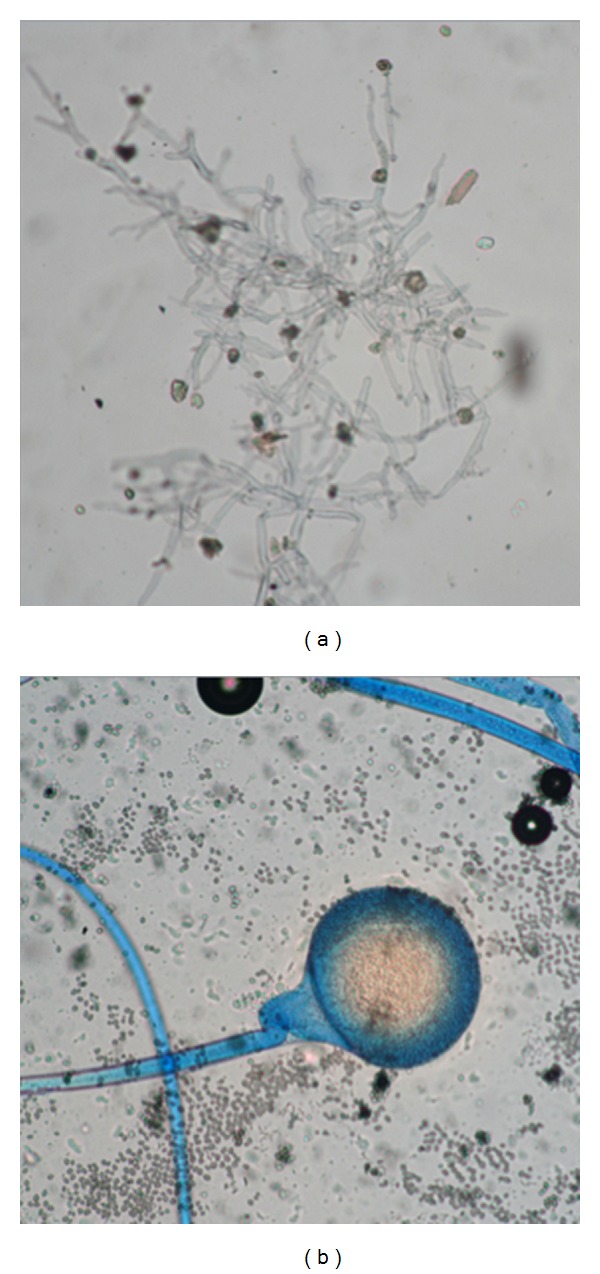
(a) KOH exam showing nonseptate hyphae branched at 90°degrees. (b) Sporangiophores with sporangia and underdeveloped stolones, compatible with *Rhizomucor sp*.

Patient no. 2 was a 54-year-old female with uncontrolled diabetes mellitus who was referred to our institution from a regional hospital with a 4-month history of fatigue, malaise, petechiae, and ecchymoses in the lower extremities. Due to the initial presumptive diagnosis of idiopathic thrombocytopenic purpura (ITP), she had been previously treated with prednisone 50 mg/d and several blood packages and platelet transfusions used. When a trend towards pancytopenia was identified, she was referred with the diagnosis of aplastic anemia. On arrival her vital signs were unstable, she was found to have facial asymmetry, periorbital edema, and bilateral proptosis (mainly right eye) with ecchymosis of the surrounding skin, her right pupil did not react to light stimuli (Figure 4). She had epistaxis along with multiple large ecchymosis and petechiae all over her body. Her laboratories showed 1.0/mm^3^ leucocytes with 0.8/mm^3^ polymorphonuclear cells, hemoglobin level of 7 gr/dL, platelet count 18.0/mm^3^; glucose 363 mg/dL, serum creatinine 1.51 mg/dL, BUN 17.6 g/dL, Na 140 mEq/L, K 2.3 mEq/L, albumin 2.6 gr/dL, ALT 30 UI/L, AST 49 UI/L, and bilirubin 2.88 mg/dL. She was started on ceftazidime, amikacin, and metronidazole with an insulin infusion and potassium replenishment. A peripheral blood smear, which was performed, showed some lymphocytes and neutrophils and no platelets. A bone marrow aspirate revealed a hypocellular bone marrow, with fibrosis, no evidence of malignant infiltration. A probable diagnosis of aplastic anemia under recovery was made. The evolution was unfavourable; after an urgent consultation to the Infectious Diseases Department a CT scan revealed extensive areas of necrosis of her right nostril, palate, and tongue. She was started on amphotericin B 1.5 mg/kg/d with a presumptive diagnosis of mucormycosis suggesting a prompt surgical debridement, and a slide smear and culture were taken. Unfortunately, before any option could be initiated, she developed respiratory and circulatory failure and died. Postmortem, a nasal biopsy and swab were taken and found compatible with rhinocerebral invasive zygomycosis due to the observation of nonseptate hyphae branched at 90° degrees (Figure 5(a)). Two days later a zygomycete was grown and identified as *Rhizomucor spp*. (Figure 5(b)).

## 3. Discussion

Mucormycoses are uncommon but aggressive infections caused by angiotropic fungi causing tissue necrosis, frequently lethal in immunocompromised hosts. *Mucor, Rhizopus, Absidia,* and *Rhizomucor* are the most common causal agents. These fungi are ubiquitous in the nature and are found in high organic matter and soil, but their potential virulence in the human host is very low [2, 3]. They can be acquired by inhalation, ingestion, or infecting wounds [4]. They usually affect the immunocompromised patient, poorly controlled diabetic, iron overload states or treatment with deferoxamine, and extensive burns [5]. The main sites involved in cancer patients have been lungs, sinuses, rhinocerebral, gastrointestinal tract, and soft tissue [2, 3, 6], but rhinocerebral and pulmonary forms together with disseminated disease have the highest mortality (78–100%) [5, 6]. Mucorales characteristically invade blood vessels, causing thrombosis and infarction with necrosis and scarring. More and more invasive fungal infections are being observed in immunocompromised patients over the past 2 decades which may be explained by an increase in the number of individuals with acquired immune deficits and aggressive therapeutic modalities like indwelling catheters, potent antineoplastic or immunosuppressive interventions, and the widespread use of antimicrobial agents [7]. Recently it has been reported that the use of voriconazole prophylaxis for invasive aspergillosis in patients with hematologic neoplasias has increased the incidence of infection by mucorales [8].

Sinusitis by mucorales should be suspected in patients with fever of unknown origin, sinunasal symptoms, mucosal discoloration, or anesthetic necrotic regions of the face or oral cavity, any of these parts may contain necrotic areas that allow prompt clues to the diagnosis when transoperative studies are analyzed, including simple procedures such as KOH test (Figure 5(a)) [9]. Mucorales enter the CNS through adjacent structures, such as paranasal sinuses or eye orbit where they can mimic a lymphoproliferative disorder. Commonly such patients complain of facial or orbital pain. At physical examination some degree of proptosis, ophthalmoplegia, altered consciousness, and nasal discharge is evident. Carotid artery thrombosis may occur [10]. 

Rapid diagnosis of mucormycosis is vital for management and therapy since these infections progress rapidly. Blood cultures are invariably negative (as in Aspergillosis), and sputum cultures and bronchial washings are seldom helpful. Fungal elements are easily detected in biopsies, nasal scrapings, or sputum: wide, ribbon-like, nonseptate, hyaline hyphae with KOH or Black Chlorazol. Special histopathological stains like Gomori-Grocott and Schiff Peryodic Acid Stain should be used. Mucorales grow in 2 or 3 days, and genera are identified by morphologic characteristics of their sporangia and sporangiophores. Only 30% of cultures are positive [11]. 

Treatment should combine early aggressive surgical excision of the necrotic lesions, restoration of immune function if possible, and amphotericin B at a dose of 1–1.5 mg/kg [3, 12–14], until remission is achieved. Once the infection has been controlled, a chronic suppressive course with amphotericin B should be considered for as long as the immunosuppression persists. Among the new antifungal drugs, posaconazole supposedly has *in vivo* action against *Rhizopus* species but other new triazolic derivates such as ravuconazole and voriconazole are inactive against mucorales, just as the echinocandins [15, 16]. 

The mortality rate among cancer patients with mucormycosis is higher, in contrast with patients without an underlying malignant disease [3]. This paper showed both sides of mucormycotic involvement in hematologic patients: the first patient, even with an unfavourable prognosis due to the ALL high-risk factors, had prompt intervention (appropriate antifungal agent plus continuous surgical debridement) which radically changed the course of this infection; meanwhile, the second patient unfortunately suffered the common pitfalls associated with a delayed diagnosis invariably progressing to fatal outcome.
